# Development and application of an automated algorithm to identify a window of consecutive days of accelerometer wear for large-scale studies

**DOI:** 10.1186/s13104-015-1229-2

**Published:** 2015-06-26

**Authors:** Eileen Rillamas-Sun, David M Buchner, Chongzhi Di, Kelly R Evenson, Andrea Z LaCroix

**Affiliations:** Fred Hutchinson Cancer Research Center, Public Health Sciences, 1100 Fairview Avenue North, M3-A410, POB 19024, Seattle, WA 98109-1024 USA; University of Illinois at Urbana-Champaign, Kinesiology and Community Health, 2021A Huff Hall, M/C 588, 1206 South Fourth Street, Champaign, IL 61820 USA; Department of Epidemiology, University of North Carolina, 137 East Franklin Street, Suite 306, Chapel Hill, NC 27514 USA; Department of Epidemiology, University of California, 9500 Gillman Drive, #0725, La Jolla, CA 92093-0725 USA

**Keywords:** Algorithm, Accelerometer, Wear window, Physical activity

## Abstract

**Background:**

Some accelerometer studies ask participants to document in a daily log when the device was worn. These logs are used to inform the window of consecutive days to extract from the accelerometer for analysis. Logs can be missing or inaccurate, which can introduce bias in the data. To mitigate this bias, we developed a simple computer algorithm that used data within the accelerometer to identify the window of consecutive wear days. To evaluate the algorithm’s performance, we compared how well it agreed to the window of days identified by visual inspection and participant logs.

**Findings:**

Participants were older women (mean age 79 years) in a cohort study that aimed to examine the relationship of objective physical activity on cardiovascular health. The study protocol requested that participants wear an accelerometer 24 h per day over nine calendar days (to capture seven consecutive wear days) and to complete daily logs. A stratified sample with (n = 75) and without (n = 100) participant logs were selected. The Objective Physical Activity and Cardiovascular Health (OPACH) algorithm was applied to the accelerometer data to identify a window of up to seven consecutive wear days. Participant logs documented dates the device was first put on, worn, and removed. Using pre-established guidelines, two independent raters visually reviewed the accelerometer data and characterized the dates representing up to seven consecutive days of 24-h wear. Average agreement level between the two raters was 90%. The percent agreement was compared between the three methods. The OPACH algorithm and visual inspection had 83% agreement in identifying a window with the same total number of days, if one or more shifts in calendar dates were allowed. For visual inspection vs. logs and algorithm vs. logs, this agreement was 81 and 74%, respectively.

**Conclusion:**

The OPACH algorithm can be efficiently and readily applied in large-scale accelerometer studies for the identification of a window of consecutive days of accelerometer wear. This algorithm was comparable to visual inspection and participant logs and might provide a quicker and more cost-effective alternative to selecting which data to extract from the accelerometer for analysis.

Trial Registration: clinicaltrials.gov identifier: NCT00000611

**Electronic supplementary material:**

The online version of this article (doi:10.1186/s13104-015-1229-2) contains supplementary material, which is available to authorized users.

## Findings

### Background

Research protocols that use accelerometers to objectively measure physical activity may ask participants to wear the accelerometer for N consecutive days. On the days the device is worn, participants are asked to document in a log when the accelerometer was put on and taken off [1, 2]. These logs provide the window of wearing days and note general periods of wear and non-wear within each day. In contrast to logs, computer algorithms, such as the one proposed by Choi et al. [3, 4], is commonly applied to the data to characterize times the accelerometer was not worn. Data from days with pre-specified amounts of wear time (e.g., at least 10 h) are then used for the statistical analysis.

Logistical and data-related challenges arise when scaling such accelerometer protocols to studies with thousands of participants [5]. One concern is participant logs can be missing or contain information that is inaccurate or incomplete. Smaller studies can ask participants to return study materials in person, providing an opportunity to review logs with the participant for completeness and accuracy. However, large-scale studies are more prone to missing logs because it is more feasible and less expensive to deliver and return study materials by mail. Missing data are usually non-random, so excluding participants with missing logs can introduce bias [6–9]. Furthermore, participants with missing logs might have worn the accelerometer. Therefore, a process is needed to extract these data from the accelerometer so that it can be included in the analysis.

**Figure 1:**
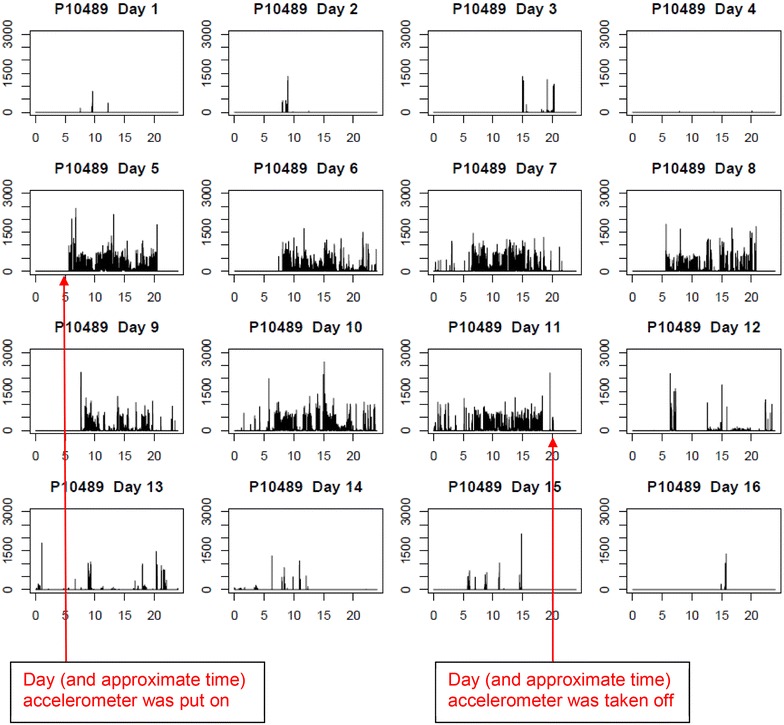
Example of an annotated accelerometer signal used during visual inspection. Each *box* represents a day, with time on the x-axis and total counts on the y-axis. Day 5 would be considered the first day and day 11 would be considered the last day of the wear window, for a maximum 7 consecutive days of wear. Day 6 would best represent the first day of wear documented in the log.

Aside from missing log data and, in particular, for protocols interested in continuous wear over N consecutive days, there might be situations when the days reported in the log do not reflect the window of consecutive days with the most analyzable data. For example, to obtain 7 days of continuous 24-h wear, up to nine calendar days of wear might be needed. On the first day, the participant puts on the accelerometer. Ideally, it is worn continuously from the second through eighth day and log data are recorded. On the ninth day, the device is removed. However, if the participant deviated slightly from this protocol, the log might not capture the window of consecutive days with the most wear. As Figure 1 demonstrates, the window from day 5 to 11 reflects 7 days, but the participant’s log (not shown) documented the window from day 6 to 12, which had 6 days.

Two potential strategies for including and maximizing the accelerometer data from missing and misinformed logs and thus, reducing bias and increasing the sample size are (1) to visually inspect the accelerometer data (“signal”) or (2) to utilize an automated algorithm. Visual inspection is not feasible in studies with large sample sizes [9]. Thus, the application of an algorithm is appealing if one can be developed with sufficient accuracy. To that end, the purposes of this study were to develop a computer-based, automated algorithm that identified a window of consecutive days of accelerometer wear and to examine how well it agreed with the window of days identified from logs and visual inspection.

## Methods

The Objective Physical Activity and Cardiovascular Health in Women (OPACH) Study, an ancillary study of the Women’s Health Initiative (WHI) Long Life Study, examines the relationship between accelerometer-measured physical activity and cardiovascular health outcomes in older women. Between March 2012 and April 2014, OPACH participants were given a hip-worn accelerometer (ActiGraph, model GT3X+) during an in-home visit or via mail. To capture seven consecutive days with 24 h of wear per day, participants were asked to wear the accelerometer on their right hip continuously over 7 days and to complete a daily sleep log (Additional file 1: Appendix A). Participants were asked to remove the accelerometer for bathing or water-based activities. Upon completion of their period of wear, all participants were asked to return study materials by mail to the coordinating center in Seattle, WA, USA. All women in the OPACH study provided informed consent and study protocols and procedures were approved by all institutional review boards (IRB) at all WHI clinic sites and institutions. The OPACH study was approved by the Fred Hutchinson Cancer Research Center IRB as an ancillary study under the WHI Extension IRB.

A total of 6,510 OPACH participants returned an accelerometer, of whom 373 (5.7%) did not return a log. For this analysis, a stratified sample of 175 OPACH participants was selected based on whether logs were available (n = 75) or missing (n = 100). However, visual inspection of the accelerometer data indicated six women from this selected sample had no or little (≤5 days) apparent wear. These six women were excluded from this analysis. This resulted in an analytic sample of 169 women, 74 with log data and 95 without.

Age at OPACH study entry, race/ethnicity, education, and self-reported physical activity were described for the sample overall and by whether they had log data. Body mass index (BMI) was calculated from height and weight clinically-measured during the OPACH home visit. Obesity was defined as a BMI ≥30 kg/m^2^. Using the WHI physical activity questionnaire [10], data about the use of assistive walking devices and engagement in physical activities, including walking, strenuous, moderate, and mild exercise, and time spent doing strenuous indoor and outdoor household chores were collected. Responses about physical activity were used to estimate energy expenditure in the form of metabolic equivalents (METS) per week [11]. Statistically significant group differences among women with and without log data were evaluated using the Chi-square test and Student’s T-test for categorical and continuous variables, respectively.

The ActiGraph GT3X+ accelerometer measures body movement on three orthogonal axes. During bodily movement, raw data from two available frequency filters (normal and low) are processed into counts per unit of time or “epoch”. The availability of two frequency filters was a feature from ActiGraph that allowed the use of different acceleration thresholds to capture and record movement. The OPACH study collected raw data in 15-s epochs using a sample frequency of 30 Hz from the normal frequency filter. The OPACH protocol was intended for the accelerometer to measure participants’ physical activity continuously over seven consecutive days. Therefore, the computer algorithm was designed to identify windows with seven consecutive days of wear.

The “OPACH” algorithm was based mainly on the observation that days with a large proportion of epochs with zero counts could be used to filter out days with no or very minimal wear. We observed that normal wear of the accelerometer yielded a substantial proportion of non-zero counts. In contrast, when the accelerometer was not worn, zero counts intermingled with a few seemingly random solitary spikes were primarily observed. Based on these observations, an algorithm was developed based on the following guidelines:

1. Defining a day as midnight to 11:59 PM, identify all days within a signal that have >10% of non-zero counts.
2. If the first day has >8 h of consecutive zero counts starting at midnight, we assumed it was a day with partial wear and excluded it.
3. If the number of consecutive days with >10% non-zero counts is ≤7, stop. This range of days represents the period of maximal accelerometer wear.
4. If the number of consecutive days is >7, apply the Choi et al. algorithm for vector magnitude [3], which distinguished wear time from non-wear time. Select the 7-day wear window with the most hours of wear.

In an overwhelming majority of participants, the first step of the automated algorithm filtered out most of the non-wearing days and reduced the data to a window of six to nine consecutive days. As a consequence, the subsequent steps in the algorithm only needed to be performed over a small window of days and was therefore computationally quick to execute. The algorithm was created using R software and programming. The annotated code has been provided in a Additional file 2.

The accelerometer signals for the 169 women with sufficient amount of wear were visually inspected and the results were compared with the OPACH algorithm. In the signals, counts per 15 s per day are displayed in a highly compressed fashion, with spikes representing periods of movement (Figure 1). Before beginning their independent reviews, the two raters drafted guidelines about how to similarly characterize the patterns in the data. Then, each rater independently assessed each signal for the following: the number of days within the signal (quality check), the days the accelerometer was put on and taken off, the day that likely reflects the first day of wear reported in the sleep log, and the start and end days of the consecutive 7-day window with the most adherent wear. Figure 1 is an example of an accelerometer signal.

Results from both raters were compared. The percent agreement for each variable and average percent agreement for all variables were calculated. Disagreements were adjudicated by consensus. Once consensus was reached for all disagreements, the findings were used to compare visual inspection with the algorithm and logs.

The wear windows identified by the algorithm, visual inspection, and logs were compared. Exact agreement was defined as having the exact same window of consecutive days, including the same total number of days and the same dates of putting on and taking off the accelerometer. When the windows differed, the level of disagreement was ascertained based on the following:

1. The date the accelerometer was put on or taken off differed, but the total number of days in the selected windows was the same.
2. The date the accelerometer was put on or taken off differed and resulted in windows with different total number of days.

The percent agreement comparing each of the three approaches was calculated. We were unable to report Kappa or intra-class coefficients because of inconsistency in our rating scale.

## Results

The mean (SD) age of the sample was 78.7 (7.3) years (range 64–96) and 30% used a walking aid at least occasionally (Table 1). One-third of women were obese. Demographic and behavioral characteristics were similar when comparing women with versus without log data.

**Table 1:** Characteristics of study sample

Characteristic	Total sample N = 169	No log N = 95	Had log N = 74
	N (%) or mean (SD)	N (%) or mean (SD)	N (%) or mean (SD)
Age, mean (years)	78.7 (7.3)	77.8 (7.1)	79.8 (7.5)
White, %	86 (50.9)	44 (46.3)	42 (56.8)
Some college education, %	108 (64.7)	61 (65.6)	47 (63.5)
Body mass index, mean (kg/m^2^)	28.3 (5.8)	28.8 (5.8)	27.8 (5.9)
Obese (BMI ≥30 kg/m^2^), %	55 (32.7)	36 (38.3)	19 (25.7)
Uses assistive walking device, %	50 (30.1)	27 (28.7)	23 (31.9)
Mean MET-h from self-reported physical activity	11.2 (14.5)	12.0 (16.4)	10.2 (11.6)

For the six variables (number of days in the signal, days the accelerometer was put on and taken off, the day that represents the first day of wear n the sleep log, and the start and end days of the consecutive 7-day window with the most adherent wear) recorded by the two visual inspectors, the range of agreement was 87–95%, with an overall average agreement of 90% (Table 2). There was 90% agreement in choosing the first day of the consecutive 7-day wear window.

**Table 2:** Results of visual inspection of 169 accelerometer signals—agreement levels between two independent raters

	Day accelerometer was put on	Day accelerometer was taken off	1st day of wear window	Last day of wear window	Day that is day 1 in the log	Average overall agreement across all items
	N (%)	N (%)	N (%)	N (%)	N (%)	N (%)
Agree	160 (95)	147 (87)	152 (90)	151 (89)	158 (94)	152 (90)
Disagree—no to little impact on data^a^	7 (4)	20 (12)	14 (8)	16 (9)	9 (5)	14 (8)
Disagree—major impact on data^b^	2 (1)	2 (1)	3 (2)	2 (1)	2 (1)	2 (1)

^a^Window of consecutive days of wear for analysis differed by 0–1 day between raters.

^b^Window of consecutive days of wear for analysis differed by 2 or more days between raters.

Among the sample of 169 participants, only comparisons between the OPACH algorithm and visual inspection could be made because log data were missing for 95 women. There was 55% (n = 93) exact agreement between the algorithm and visual inspection (Table 3). In 27% (n = 46) of all the participants, these two methods identified wear windows with the same total number of days, but had a 1-day difference in the calendar dates, which resulted in 18 more minutes of wear by visual inspection. In sum, there was 83% (n = 141) agreement in the selection of a wear window with the same total number of days, if one or more differences in the calendar dates were allowed in the calculation. For 28 (17%) signals, the number of days in the selected wear windows differed, with the algorithm identifying fewer days of wear than visual inspection.

**Table 3:** Comparison of algorithm, visual inspectio n, and logs in the identification of the window of consecutive days of wear

	Total sample n = 169	Sample with logs n = 74	
	Visual inspection vs. algorithm N (%)	Visual inspection vs. logs N (%)	Algorithm vs. log N (%)
Same number of days in wear window			
Complete agreement	93 (55.0)	50 (67.6)	39 (52.7)
Wear window shifted by 1 day in one method	46 (27.2)	10 (13.5)	15 (20.3)
Average difference in hours of wear	0.3 (more in visual inspection)	5.3 (more in visual inspection)	1.5 (more in algorithm)
Wear window shifted by ≥2 days in one method	2 (1.2)	0 (0)	1 (1.4)
Average difference in hours of wear	2.2 (more in algorithm)	N/A	4.5 (more in algorithm)
Total, N (%)	141 (83.4)	60 (81.1)	55 (74.3)
Different number of days in wear window			
Differed by 1 day	25 (14.8)	13 (17.6)	19 (25.7)
Method with more days of wear	Visual inspection	Visual inspection	See footnote^a^
Differed by ≥2 days	3 (1.8)	1 (1.4)	0 (0)
Method with more days of wear	Visual inspection	Visual inspection	N/A
Total, N (%)	28 (16.6)	14 (18.9)	19 (25.7)

^a^Algorithm had more days for 9 (12.2%) signals; log had more days for 10 (13.5%) signals.

Table 3 also shows the agreement levels of the OPACH algorithm and visual inspection compared to the logs for the 74 women with non-missing log data. Relative to visual inspection and the algorithm, there was 68% (n = 50) and 53% (n = 39) exact agreement, respectively, with the log in the selection of the consecutive 7-day wear window. These percentages increased to 81% (n = 60) and 74% (n = 55), respectively, if one or more shifts in calendar dates were allowed, but the total number of days remained the same. Between visual inspection and the sleep logs, 19% (n = 14) of signals had different numbers of days in the wear windows, with visual inspection having more wear days in all cases. Between the algorithm and sleep logs, 26% (n = 19) signals had different numbers of days in the wear window, but the algorithm chose more wear days in 12% (n = 9) of the signals, while the sleep log chose more wear days for 14% (n = 10) of the signals.

## Discussion

We developed a simple automated algorithm to identify a window of consecutive days of accelerometer wear for the extraction of analyzable accelerometer data. This algorithm can easily be modified and tailored to other accelerometer study protocols so that researchers can readily apply to their own accelerometer data. The use of an algorithm to identify the window of accelerometer wear days has great appeal in studies with large sample sizes. Operationally, the algorithm can be applied to large amounts of data and is computationally quick to execute. In OPACH, we applied the algorithm to (1) increase sample size because it allowed us to include accelerometer data from women with missing logs and to (2) reduce bias because we were able flag discrepant data and check the quality of the self-reported log data. However, other applications of the OPACH algorithm might also include (1) identifying a wear window with N consecutive days from the accelerometer, (2) assisting in the cleaning of visually inspected data or, (3) improving data quality (e.g. triangulating visual inspection, algorithm, and logs data to optimize the amount of analyzable accelerometer data).

The level of agreement comparing visual inspection, algorithm, and logs was similar. Exact agreement was fair (53–68%) and there was good agreement (74–83%) in identifying windows with the same total number of days. When one method identified more days than another, it almost always identified exactly one additional day. The agreement among methods was over 98% for identifying windows with either the same total number of days or a difference of exactly 1 day.

One explanation of these findings begins with the observation that virtually all accelerometer signals in this study contained one cluster of consecutive wear days. Uniformly, each method identified the cluster within their wear windows. The OPACH study protocol asked participants to wear the accelerometer over 9 calendar days and the majority of women wore it for all of this time. The unanticipated consequence was that any day of wear could be regarded as an adherent wear day. Since adherence of more than 7 days can yield multiple 7-day wear windows, identifying the same number of days within the wear window was achievable even when the methods selected different start or end days. Alternatively, because our sample had good adherence, it is unclear whether we would observe the same agreement levels in participants with lower adherence.

Each method had different rules for choosing the window with the most days, resulting in varying levels of agreement. The log did not allow participants to record days outside of the second through eighth day of wear. Data in logs can have error and studies have reported that logs overestimate wear time compared to accelerometer-based algorithms that determine non-wear time [7, 9]. Visual inspection sometimes struggled to classify wear versus noise and to estimate whether the number of hours of wear per day exceeded the minimum required. Others have noted that visual review of accelerometer data might be prone to misclassification of sedentary behavior as non-wear [7]. On a partial wear day that included both wear time and artifact, the algorithm added together the counts from both movement and artifact to establish whether the day was an adherent day.

Taken collectively, we interpreted the results to indicate that it might be worthwhile to use a combination of visual inspection, logs, and algorithms to identify the optimal window of wear days for data analysis. Each method identified different numbers of adherent days in 15–26% of participants. Thus, it is possible that each method, when used alone, might exclude a different subgroup of participants. Hence, we believe it might be more suitable to use data from all three methods to identify the window of accelerometer wear days for participants. In OPACH, both the algorithm and logs are used to identify the window of consecutive days for data analysis in participants with five or more adherent days, but uses all three methods for participants with four or less adherent days.

There are limitations to this study. The results reported are specific to the OPACH study protocols, which can be improved. For example, logs could be designed to document wear whenever it occurs rather than over a consecutive 7-day window. Additional testing might yield an automated algorithm with higher accuracy. Second, there are advantages to using a data processing protocol that prioritizes choice of the analysis window based upon the log. For example, OPACH preferred the window from the log data unless the algorithm identified a window with more adherent days. This implies that the self-reported log has the most useful information. Yet, it is possible that no wear was documented during a period of time that the accelerometer showed wear, so it would be useful to check the data in the accelerometer to determine if there are more adherent days of wear than the log indicates. The results in Table 3 suggest that, at least in OPACH, this situation may occur in roughly 25% of the participants.

Although it is possible that visual review of accelerometer signals misclassified sedentary behavior as non-wear, the tri-axial accelerometers should be more sensitive to any movement and the main objective of visual inspection was to identify days where the accelerometer was worn for any amount of time. A non-wear algorithm can be applied to these days to characterize wear time more precisely than is possible from visual inspection. Indeed, research has suggested that characterization of non-wear time is more accurate when applied to tri-axial accelerometer data compared to uniaxial [4].

Finally, the study was not able to summarize agreement using kappa and intra-class correlation coefficients because of the complex nature of the rating task (i.e., the comparison of multiple consecutive 7-day windows). These statistical measures assume the same scale is used for each rating, such as “yes versus no” or a 100 point scale. However, because accelerometer signals contained a variable number of days, the rating scale varied. For example, in a 12-day signal with 6 days of wear, the possible first days of wear are day one through day seven, so the rating was on a seven-point scale. However, in a 30-day signal and 6 days of wear, the possible first days of wear are day one thru day 25, yielding a 25-point scale. That is, the probability of chance agreement of raters varied by the number of days recorded in the signal. Furthermore, the kappa coefficient only distinguishes agreement and disagreement and does not quantify the magnitude of disagreement. For example, comparing two 7-day windows that differ by a 1-day shift results in a 6-day overlap, but a difference by a 7-day shift would result in no overlap of days. These two scenarios would both be regarded as disagreement in the kappa coefficient calculation, yet suggest different levels of agreement: good, but not complete agreement in the former scenario and poor agreement in the latter scenario.

The development and use of algorithms in accelerometer studies is common. Applications of automated algorithms include distinguishing times of wear and non-wear [3, 4, 7, 9, 12], characterizing sedentary time [13, 14], and reducing data for processing and summarizing [6, 8]. This study contributes another application—that of identifying a window with N consecutive days of accelerometer wear. Although the OPACH algorithm can be used in its current form, we recommend that the tuning parameters (e.g., percent threshold for non-zero counts, minimum daily hours of wear, total consecutive days) are modified for other studies with different protocols and cohort characteristics. About 6% of OPACH participants had missing logs. The algorithm allowed inclusion of these women in the analysis, thus decreasing bias and increasing statistical power. For large-scale studies, an automated algorithm is an efficient approach for determining what data to extract from the accelerometer for analysis. Additionally, it is cost-effective since it reduces both researcher and participant burden in the collection, cleaning, and processing of logs.

## Conclusion

We developed a simple automated algorithm that can be efficiently and readily applied to large amounts of accelerometer data for the identification of a window of consecutive days of accelerometer wear. This algorithm was comparable to visual inspection—a method not feasible in large-scale studies—and self-reported logs—the standard method of accelerometer data extraction in most studies of objective physical activity—and might provide a quicker and more cost-effective alternative to extracting data from the accelerometer for analysis.

## Availability and requirements

The R-code for the execution of the OPACH algorithm has been provided in Additional file 2. The R program is needed to execute the code as is. The code may be modified to execute in other statistical software packages.

## Availability of supporting data

This is not applicable to this study.

## Additional files

Additional file 1: OPACH daily sleep log. Additional file 2: R code for execution of OPACH algorithm.

## Abbreviations

BMI: body mass index
IRB: institutional review board
OPACH: Objective Physical Activity and Cardiovascular Health in Women
WHI: Women’s Health Initiative
