# Supplementary material for: Development and application of an automated algorithm to identify a window of consecutive days of accelerometer wear for large-scale studies
Source: BMC Res Notes. 2015 Jun 26;8:270. doi: 10.1186/s13104-015-1229-2 (PMC4482153; doi:10.1186/s13104-015-1229-2)
Supplement: Additional file 1: — OPACH daily sleep log. [file 13104_2015_1229_MOESM1_ESM.docx]

**Please complete this form each day for the next 7 full days you are wearing the activity monitor.**

**Instructions:**

- **Start recording on the day the monitor is put on, which is also your appointment day. That night, record the time you went to bed for the night.**
- **Then, each day for 7 full days, please record the time you got out of bed and the time you went to bed.**
- **After wearing the monitor for 7 full days, please remove it. On that day, you only need to record the time you got out of bed.**
- **When you have removed the monitor, please send the monitor, the belts, this form, and *Form 321 – Physical Activity Questionnaire* back to the study center in the envelope provided.**

| **Day** | **Date** | **Time out of bed in the morning** | **Time into bed for the night** | **List any times the monitor was not worn and state reason for not wearing it (e.g., bathing).**  **Any additional comments?** |
| --- | --- | --- | --- | --- |
| **Day Monitor Put On** | __ __ / __ __ / __ __  MM DD YY | *Not applicable* | □ am __ __:__ __ □ pm  Hr Min |  |
| **1** | __ __ / __ __ / __ __  MM DD YY | □ am __ __:__ __ □ pm  Hr Min | □ am __ __:__ __ □ pm  Hr Min |  |
| **2** | __ __ / __ __ / __ __  MM DD YY | □ am __ __:__ __ □ pm  Hr Min | □ am __ __:__ __ □ pm  Hr Min |  |
| **3** | __ __ / __ __ / __ __  MM DD YY | □ am __ __:__ __ □ pm  Hr Min | □ am __ __:__ __ □ pm  Hr Min |  |
| **4** | __ __ / __ __ / __ __  MM DD YY | □ am __ __:__ __ □ pm  Hr Min | □ am __ __:__ __ □ pm  Hr Min |  |
| **5** | __ __ / __ __ / __ __  MM DD YY | □ am __ __:__ __ □ pm  Hr Min | □ am __ __:__ __ □ pm  Hr Min |  |
| **6** | __ __ / __ __ / __ __  MM DD YY | □ am __ __:__ __ □ pm  Hr Min | □ am __ __:__ __ □ pm  Hr Min |  |
| **7** | __ __ / __ __ / __ __  MM DD YY | □ am __ __:__ __ □ pm  Hr Min | □ am __ __:__ __ □ pm  Hr Min |  |
| **8** | __ __ / __ __ / __ __  MM DD YY | □ am __ __:__ __ □ pm  Hr Min | *Not applicable* |  |
